# Enhancing antimicrobial resistance (AMR) surveillance data use in Uganda

**DOI:** 10.1017/ash.2025.10264

**Published:** 2026-02-23

**Authors:** Uzo Chukwuma, Jonathan Mayito, Vivian Twemanye, Ritah Namusoosa, Dan Kakyakumaiso, Morgan Otita, Dickson Tabajjwa, Consolata Guma, David Brett-Major, Richard Walmema

**Affiliations:** 1 Department of Epidemiology, College of Public Health, University of Nebraska Medical Center, Omaha, NE, USA; 2 Division of Epidemiology and Disease Prevention, Indian Health Service, Rockville, MD, USA; 3 Department of Global Health Security, Infectious Diseases Institute, Makerere University, Kampala, Uganda; 4 National Microbiology Reference Laboratory, National Health Laboratory and Diagnostic Services, Ministry of Health, Kampala, Uganda; 5 Jinja Regional Referral Hospital, Jinja, Uganda

## Abstract

**Objective:**

This study aimed to identify gaps and barriers to the use of antimicrobial resistance (AMR) surveillance data in Uganda and to recommend enhancements to improve policy and intervention outcomes.

**Design:**

A comprehensive assessment was conducted within a framework of infrastructural and system analysis, stakeholder engagement, and data management simulations.

**Methods:**

The assessment consisted of three components: (1) assessing data utilization through stakeholder engagement to identify barriers in data translation and use; (2) evaluating the existing infrastructure and surveillance system supporting AMR data flow; and (3) simulating processes of data management and flow to contextualize identified gaps.

**Results:**

Findings revealed deficiencies in the AMR data governance structure and informatics capabilities. Stakeholders highlighted limited access to data and analytical capacity as barriers to effective decision-making. The existing infrastructure lacks the capability for real-time data analysis, which limits the ability to inform national and health facility policies. Strengthening data management processes, enhancing analytical tools, and fostering stakeholder collaboration at all levels are recommended for efficient data utilization.

**Conclusions:**

Addressing these gaps is crucial for strengthening AMR surveillance in Uganda, enabling more effective data use to guide intervention and policies, and ultimately improving public health outcomes.

## Introduction

Antimicrobial resistance (AMR) is a global health crisis, projected to cause 10 million deaths annually by 2050 if unaddressed.^1^ A 2021 global review found 1.14 million deaths directly attributed to AMR, 4.71 million associated deaths, and an 80% rise in mortality among adults aged 70 years and above from 1990 to 2021.^2,3^ These trends underscore the need for strengthened systems and strategies that support effective, data-driven AMR policies and interventions.

The burden of AMR falls disproportionately on low- to middle-income countries.^4^ Sub-Saharan Africa reports the highest AMR-attributable death rate at 27.3 per 100,000 people,^3^ worsened by inadequate surveillance systems.^5^ In 2018, in response to the AMR threat, Uganda developed a National Action Plan on AMR and a One Health (OH) Strategic Plan, both employing an OH approach to prevent emergence and spread of AMR.^6,7^ These plans outline a roadmap for addressing AMR containment infrastructure and capacity gaps in the country.

Uganda’s efforts to improve infrastructure and capability deficits involved leveraging external catalytic grants such as the Fleming Fund Country and Regional grants.^8^ Uganda has made notable gains in addressing these gaps; however, deficits in data utilization for clinical practice and policy remain. Fragmented data systems, reliance on paper-based processes, limited feedback mechanisms, and insufficient analytical capacity continue to hinder the translation of data into actionable insights.

Uganda participated in the World Health Organization Joint External Evaluation in 2017 and 2023 to identify capacity gaps that impact response to public health threats.^9^ Both evaluations highlighted the need to strengthen real-time AMR surveillance, data analysis, information sharing, and optimal use of antimicrobials.^9^ Additionally, a Fleming Fund unpublished report further identified gaps in surveillance capacity, routine data analysis for outbreak detection, and limited integration of AMR surveillance into infection prevention and control practices. This report emphasized the need for improved data cleaning, sharing, and dissemination. This aligns with research showing that limited data management and analytical capacity are critical barriers to optimal AMR data use and undermine effective responses to the threat. ^10^

Despite having infrastructure for governance, laboratories, and data collection, Uganda still struggles to translate AMR data effectively. Identifying barriers to effective data utilization is crucial for developing solutions and implementation strategies. This study assessed the gaps in Uganda’s AMR surveillance system that limit data utilization. Using system assessment, stakeholder engagement, and data flow simulations, it identified critical barriers to translating surveillance data into action and proposed practical solutions.

## Methods

The study methods consisted of three components: stakeholder engagement and data utilization, surveillance system and infrastructure review, and process simulation of data management and flow. Together, these assessments provided a framework for systematically identifying barriers and generating practical recommendations. These stakeholders were engaged throughout all three components of the assessment. National-level stakeholders included the National Microbiology Reference Laboratory (NMRL) and the National Coordination Center (NCC) for AMR surveillance. Regional-level stakeholders were engaged through a case study at Jinja Regional Referral Hospital (RRH), representing the broader RRH network. Participants included an infectious disease specialist, a microbiologist, a pharmacist, a pediatrician, members of the Medicine and Therapeutics Committee, the infection prevention and control subcommittee chair and regional focal person, and the head of the microbiology department. Data use was defined as transforming AMR data into actionable information for clinical, public health, and policy decisions. It was assessed across three dimensions: accessibility (timeliness and usability), translation (analysis and interpretation), and application (integration into intervention and policy).

### Data utilization assessment and key stakeholder engagement

This component assessed the current access to AMR data, utilization, and barriers to effective translation into interventions, policies, and clinical decisions. This assessment was conducted through participatory stakeholder engagement and direct observation of AMR data use. Additional national-level stakeholders specific to this component included the Makerere University Infectious Diseases Institute Global Health Security team, the National OH platform chair, and the National AMR subcommittee chair.

At Jinja RRH, one of Uganda’s primary specialist facilities, the study evaluated AMR data management practices, data utilization, and analytical capacity.

Structured discussions explored routine practices, challenges in accessing and analyzing data, and specific decision-making contexts in which data were not effectively applied. Document reviews supplemented these engagements to identify policies informed by data generated within Uganda’s AMR infrastructure. These assessments revealed where and why data-use gaps occur and how they hinder translation into action.

### Surveillance system and infrastructural assessment

This component evaluated the structural and technological foundations of AMR surveillance. This included reviewing existing informatics applications for data management, analytics, decision-making, and operational efficiency, with a focus on AMR data flow from the regional referral hospitals to the national levels. Analytical capacity was evaluated by examining available data management and analysis tools, analytical capabilities, and processes for real-time data access. The study also assessed the infrastructure and workflow needed to generate decision-making products for stakeholders.

At the regional referral hospital, laboratory information systems such as the African Laboratory Information System (ALIS) complement paper and electronic medical record systems, such as Clinic Master and EAFYA, to support AMR data management. ALIS, a laboratory information system, manages clinical workflows and AMR data, while the World Health Organization Network (WHONET), an epidemiological analysis application, supports management and interpretation of AMR data. ClinicMaster and EAFYA capture and integrate healthcare information for patient care and outcomes.

### Process simulation

The final component involved simulating AMR data flow using sample patient datasets. The simulation tested the processes described by stakeholders and observed in the infrastructure assessment, allowing replication of how data is generated, transmitted, analyzed, and used in practice for ALIS and WHONET users. The process simulation assessment compared current surveillance functions to WHONET capabilities. The study considered solutions beyond WHONET; however, the applicability of these solutions in resource-constrained settings was given greater weight. This study did not involve research on human subjects.

## Results

Table 1 outlines the alignment of Uganda’s AMR strategies, evaluations, and grant support, highlighting progress and gaps in surveillance and data use. It synthesizes objectives from the National Action Plan, OH Strategic Plan, Fleming Fund activities, and World Health Organization Joint External Evaluation scores (2017, 2023). This table illustrates the country’s strategic emphasis on infection prevention, antimicrobial stewardship, and surveillance in its AMR priorities and objectives.

**Table 1.**
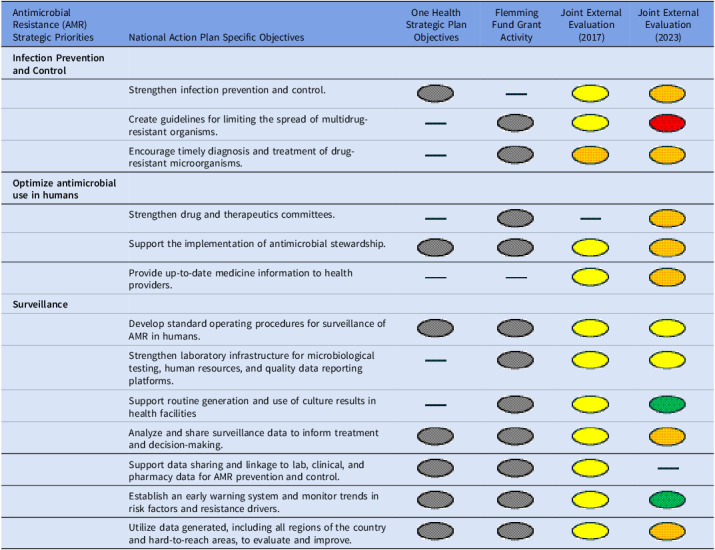
AMR priorities, objectives, grant activities, and external evaluations

Notes. The evaluation scores are color-coded: The grey circle represents present and included. The green circle represents a score of 4 or 5 (demonstrated or sustainable capacity). The yellow circle represents a score of 3 (developed capacity). The orange circle represents a score of 2 (limited capacity). The red circle represents a score of 1 (no capacity).

Dash (—) indicates no data or information was reported for that cell.

### Overview of governance and informatics structure

At the national level, Uganda’s AMR governance is coordinated through the OH platform, the highest-level coordinating body, integrating national and regional structures, key ministries, AMR subcommittees, technical working groups, and partners. The National AMR Subcommittee, under this OH platform, oversees implementation of the AMR action plan through its technical working committees. The technical working groups also focus on developing policy and strategy.^11^ The national infrastructure includes administrative oversight under the Ministry of Health’s Department of National Health Laboratory and Diagnostic Services and the structural entities of the NMRL and the NCC, all housed within the Central Public Health Laboratory. The NMRL, a stand-alone reference microbiology laboratory, conducts quality checks and assurance of AMR data from regional hospitals and augments laboratory capacity across the country. The NCC manages national reporting and dissemination. The regional infrastructure, represented by the RRH, serves as the first level within the tiered healthcare system with laboratory and clinical patient care capacity. AMR data utility at the regional facility includes informing clinical decisions, tailoring treatment plans, developing facility-wide policies, and promoting the rational use of medicines and therapeutic interventions.

### AMR surveillance systems and data flow at the regional level – regional referral hospital

RRHs in Uganda use a paper-based system for initial patient screening, guiding triage, and implementing infection control protocols. Clinicians use a paper-based microbiology form to request tests and capture crucial AMR data, including clinical information, laboratory identification number, unique patient identification, type of sample, and type of specimen. Each clinical visit generates a unique patient identification number linked to either the inpatient or outpatient departments. The unique identification number does not align with the national identification number. The national identification number is not integrated into clinical services systems.

**Figure 1.**
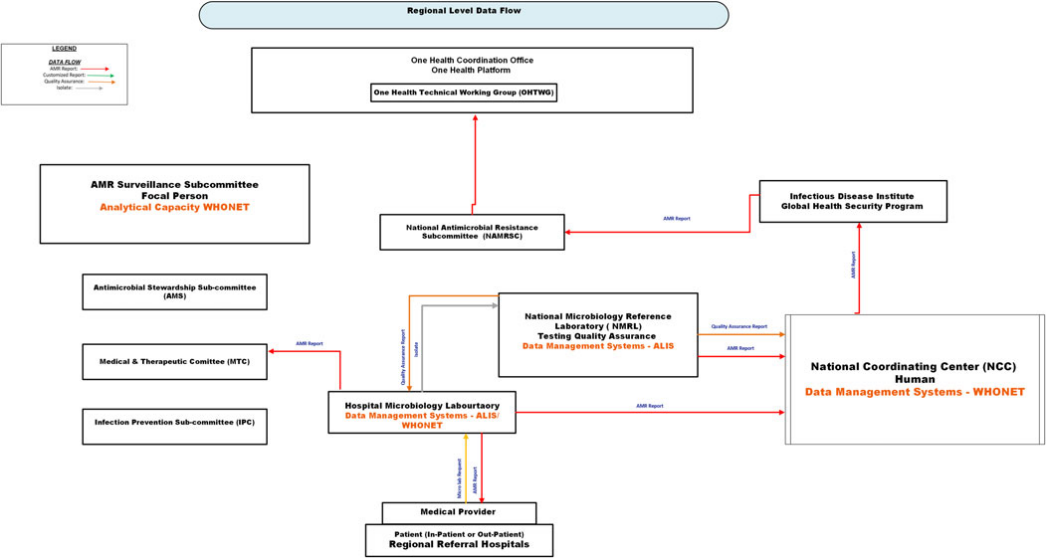
Current regional-level antimicrobial resistance (AMR) data flow in Uganda. Solid arrows represent key information flows: red = AMR surveillance report from World Health Organization Network (WHONET) or the African Laboratory Information System (ALIS); green = customized report; orange = quality assurance feedback; grey = physical transfer of isolates. Data originates at the patient-provider interface and is captured at the hospital microbiology laboratory using ALIS or WHONET. AMR data and isolates are routed to the National Microbiology Reference Laboratory (NMRL) for testing and quality assurance, and to the National Coordination Center (NCC) for national analysis and reporting. The NCC, upon request, can share national AMR data with the Infectious Disease Institute (IDI) and routinely provide it to the National Antimicrobial Resistance Subcommittee and the One Health Technical Working Group (OHTWG). Within the facility, AMR data from the microbiology laboratory are utilized by the AMR Surveillance Subcommittee and clinical governance bodies, including the Antimicrobial Stewardship Subcommittee (AMS), the Medicine and Therapeutics Committee (MTC), and the Infection Prevention and Control Subcommittee (IPC), to inform decision-making. ALIS, African Laboratory Information System; WHONET, World Health Organization Network; NMRL, National Microbiology Reference Laboratory; NCC, National Coordination Center; IDI, Infectious Disease Institute; OHTWG, One Health Technical Working Group.

Figure 1 illustrates the current flow of AMR data in the regional referral hospital. Microbiology laboratories manage AMR data by submitting ALIS files to the NCC or transcribing data into WHONET for submission. Both methods capture test requests and antimicrobial susceptibility testing (AST) results, which are stored in ALIS or WHONET. Feedback within the regional referral hospital relies on a paper system and email. The AST results are either printed or handwritten on a result form, which clinicians manually collect from the pigeonhole in the laboratory and submit quarterly Microsoft Excel® reports to NMRL.

WHONET enhances analytical capacity by generating decision-support products, such as antibiograms, line lists of resistant pathogens, and outbreak alerts, organized by hospital location and resistance profile. AMR data from ALIS is exported to Excel and then converted through BacLink for WHONET analysis.

The microbiologist, biostatistician, or AMR focal person disseminates aggregated AMR data and supports hospital subcommittees (such as antimicrobial stewardship, infection prevention and control, and medicine and therapeutics) with analytics and decisional products via paper or email. The infection prevention and control focal person coordinates infection control activities across the hospital and region, using a paper-based system to share AMR information. All RRHs must submit their AMR data to the NCC; WHONET users email their exports, and ALIS users email ALIS files. Patient isolates are also sent to NMRL for quality assurance.

### AMR surveillance systems and data flow at the National Level—Ministry of Health—Central Public Health Laboratory

#### National Microbiology Reference Laboratory

**Figure 2.**
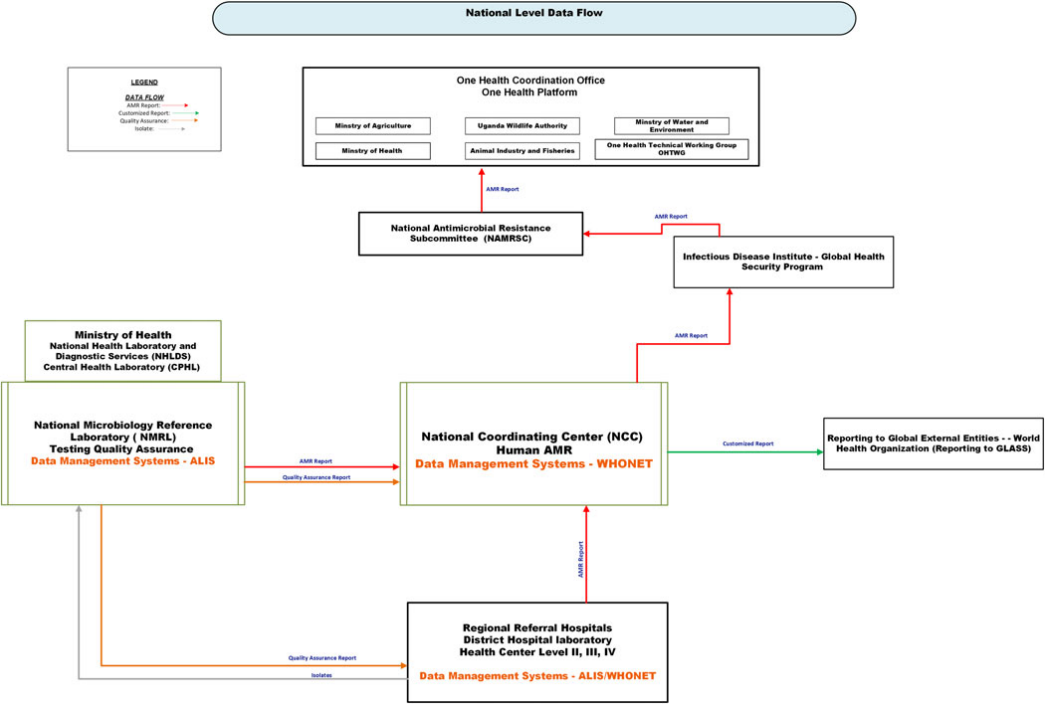
Current national-level antimicrobial resistance (AMR) data flow in Uganda. The Figure depicts the flow of AMR data, quality assurance feedback, and bacterial isolates among national stakeholders. Solid arrows indicate the direction of information flow: red = AMR surveillance reports from World Health Organization Network (WHONET) or the African Laboratory Information System (ALIS); green = customized reports; orange = quality assurance reports; grey = physical transport of isolates. AMR data generated at regional referral hospitals and lower-level health facilities are captured through ALIS or WHONET and transmitted to the National Coordination Center (NCC) for national analysis and reporting to external partners, such as the World Health Organization, via GLASS (Global Antimicrobial Resistance and Use Surveillance System). The NCC, upon request, can share national AMR data with the Infectious Disease Institute (IDI) and routinely provide it to the National Antimicrobial Resistance Subcommittee and the One Health Technical Working Group (OHTWG). The National Microbiology Reference Laboratory (NMRL) provides confirmatory testing and quality assurance for laboratory data from the facility. The Ministry of Health provides oversight through the Central Public Health Laboratory (CPHL), which is part of the National Health Laboratory and Diagnostic Services (NHLDS). ALIS, African Laboratory Information System; WHONET, World Health Organization Network; NCC, National Coordination Center; IDI, Infectious Disease Institute; OHTWG, One Health Technical Working Group; NMRL, National Microbiology Reference Laboratory; GLASS, Global Antimicrobial Resistance and Use Surveillance System; CPHL, Central Public Health Laboratory; NHLDS, National Health Laboratory and Diagnostic Services.

Figure 2 illustrates the current national AMR data flow. The core function of the NMRL is to conduct quality assessments of isolates, support diagnostics for multidrug-resistant pathogens, and enhance laboratory capacity for lower-level laboratories. NMRL requires regional laboratories to send all or a portion of their isolates for quality assurance. Feedback is provided to submitting laboratories via email to support AMR interventions, clinical care, and testing quality improvement.

NMRL manages data using ALIS and analyzes it with WHONET as needed. Isolates for quality assurance are accompanied by identifying variables recorded on paper. NMRL re-accessions each isolate, generating a new identification number that is stored in ALIS alongside the original. AST results are emailed to NCC for AMR analysis and reporting.

#### National Coordination Center

The NCC serves as the national hub for managing and analyzing AMR data files from the regional level, supporting quality assurance and policy. It utilizes WHONET for data management and analysis and coordinates with NMRL to perform quality assurance on data submitted from the regional level. NCC receives WHONET or ALIS AMR data exports via email, with ALIS exports requiring BacLink conversion before analysis in WHONET. NCC produces facility antibiograms, fulfills ad hoc data requests, offers WHONET training, and manages national data reporting.

### Key barriers to data utilization at the regional and national level

Table 2 highlights key barriers to regional and national AMR surveillance and data use, including reliance on paper-based systems, email communication systems, and limited automated processes.

**Table 2.** Gaps in the AMR surveillance structure at the regional and national level

Gaps Identified at the Regional Level
1. AMR data related to sample collection is recorded using a paper-based microbiology laboratory request form.
2. Critical elements on the microbiology laboratory request form are not standardized, and there is no national system for generating uniquepatient identification; each facility uses its own method. Required fields on the paper form cannot be consistently enforced.
3. Applications for managing AST results vary between ALIS and WHONET, but ALIS is not functional at all RRHs.
4. ALIS lacks analytical functionality and cannot interoperate with WHONET for data transfer. Additionally, there is limited knowledge of how to convert ALIS data to WHONET.
5. The data preparation process for WHONET analysis is not standardized nationally, and users continue to face challenges in using its analytical functions.
6. No standardized decision-making tools have been established.
7. The regional level lacks both a timely alert system to notify stakeholders and a feedback mechanism to share decision-making products.
8. Interconnectivity between the systems that track AMR information—clinic master, ALIS, and WHONET does not exist.
9. Improve data flow and timely access to decision-making products by establishing efficiencies; process automation is not in place.
10. There is no centralized repository of AMR data accessible to all stakeholders for informing policy, advocacy, and intervention planning.
**Gaps Identified at the National Level**
1. At the national level, there is no interoperability between ALIS and WHONET, which impacts the transfer of AST results to WHONET for analytics.
2. The ALIS application used at the national and regional levels is not interoperable.
3. Training is needed in preparing data for conversion and analysis.
4. Feedback on quality assurance reports is often delayed.
5. Regional-level AMR data exports from ALIS are manually processed before national-level consolidation. Automated consolidation is needed.
6. Analytical capacity for reconciling all AMR data collected is necessary. The lack of a unique identifier for each patient could impact national-level analytics.
7. Limited analytical capacity to support ad-hoc requests from facilities, special reports for stakeholders, and national reports.
8. There is no centralized national repository of AMR data accessible to all stakeholders for informing policy, advocacy, and interventions.

Notes. ALIS, African Laboratory Information System; AMR, Antimicrobial Resistance; AST, Antimicrobial Susceptibility Testing; RRH, Regional Referral Hospital; WHONET, World Health Organization Network.

Laboratory test request forms are paper-based, and AST results are sent to clinicians via email or paper logs, which impact timely access to the data in an efficient, usable format. The inconsistent method for generating unique patient identification (limited use of national identification numbers and facility-generated identification numbers per visit) impedes data translation by preventing accurate linkage across visits, complicating analysis and interpretation, and reducing overall efficiency in tracking patient outcomes.

AMR data management is fragmented across ALIS and WHONET and does not interface with the electronic medical records systems. Interoperability challenges and manual processes in formatting data from ALIS to WHONET delay analysis and limit its use for timely decision-making and policy development. Facilities using WHONET can generate decision-making products; however, the absence of standardized data preparation methods compromises the reliability for clinical application, policies, and comparability.

The stakeholder discussions during the data utilization assessment corroborated findings from the 2023 surveillance assessment and the unpublished Fleming Fund report, revealing that AMR data and decisional reports were not readily accessible to support policies, interventions, or clinical decisions. Overall, reporting to stakeholders is inefficient because it relies on paper or email to share data, given the non-interoperable nature of regional and national systems. Facilities lack an intranet or internal communication systems, further delaying the integration of data for interventions. Paper-based systems also hinder real-time infection prevention and control efforts. Stakeholders also noted the lack of robust feedback mechanisms, limited WHONET training, and insufficient time for data analysis as key challenges to the utilization of the data.

Table 2 outlines the gaps in AMR data flow at the national level. The email system facilitates feedback on quality assurance results from the national to the regional levels, but not in real time. Interoperability issues persist even at the national level, with no connectivity between ALIS and WHONET, requiring conversion via BacLink. There is no standardized configuration for BacLink data conversion; configurations vary among all users.

The NCC disseminates AMR reports to the One Health Platform’s committees via email. However, the NCC lacks the capacity to generate decision-making products such as antibiograms, outbreak alerts, and epidemiological trend reports, thereby limiting the utility of the data. Additionally, limited staff capacity delays response to ad hoc data requests. Stakeholders also noted that the capacity to translate the data into actionable insights hindered its application to national policy.

## Discussion

This study identified barriers to effective AMR data management and utilization in Uganda, highlighting gaps that impede translation of surveillance outputs into timely clinical and policy actions. While Uganda has demonstrated a strong commitment to AMR through its strategic objectives and plans, the regressing World Health Organization Joint External Evaluation scores between 2017 and 2023 suggest persistent systemic implementation challenges in operationalizing surveillance and data-driven decision-making.^9^ In well-functioning AMR surveillance systems, such as in Europe and Asia, standardized digital platforms, real-time data sharing, and established analytical pipelines enable timely feedback between laboratories, clinicians, and policymakers.^12,13^ In contrast, Uganda’s reliance on paper-based systems, fragmented digital infrastructure, and limited analytical capacity creates delays, inefficiencies, and missed opportunities for intervention.

The stakeholder engagement revealed that weak data flows undermine AMR data use in Uganda’s healthcare system. Reliance on paper-based processes introduces transcription errors and inconsistent data capture, undermining data quality and effective decision-making. Inconsistent patient identifiers further erode data quality, reducing confidence in surveillance outputs. AST results shared via paper or email create bottlenecks in data aggregation and quality assurance, while the lack of integration between data systems and ClinicMaster limits intra-facility communication and timely action. In contrast, more robust AMR systems use interoperable digital platforms for real-time data sharing among laboratories, clinicians, and policymakers.^14^ Without investments in interoperability and streamlined processes, Uganda’s surveillance data will remain underutilized, limiting its impact on clinical care and policy development.

Simulation of data processes confirmed stakeholder concerns, showing that interoperability challenges, particularly between ALIS and WHONET, limit efficient aggregation and analysis of data. While WHONET provides analytical functions, ALIS users struggle to transfer AST results into WHONET due to interoperability issues and limited user proficiency. Facilities using WHONET bypass these barriers but often lack the skills to prepare the data or apply advanced features, such as generating antibiograms. This gap is critical, as antibiograms guide empiric prescribing and inform stewardship interventions.^15^ The lack of tailored standard operating procedures leads to inconsistent practices, which are vulnerable to staff turnover. Furthermore, the limited time for WHONET self-training compounds the problem. In contrast, countries like India and South Africa have improved efficiency through automated data integration and standardized antibiogram generation, thereby reducing reliance on individual expertise.^16,17^ Without similar investments, analytical inefficiencies will persist, limiting the data’s utility.

Delayed and inefficient data capture compromises clinical responsiveness, increasing the risk of inappropriate or prolonged antibiotic use.^18^ National and regional health facility communication fragmentation of AST results to key stakeholders, such as infection control, medicine and therapeutics, and the antimicrobial stewardship subcommittees, limits timely interventions, highlighting the need for centralized repositories and structured reporting pathways. Table 3 highlights practical recommendations for improvements. Implementing standardized data management systems, electronic laboratory requests, uniform sample collection, and unique patient identifiers can enhance data quality and timeliness. These strategies align with global best practices, where automated surveillance and standardized reporting enable rapid translation of laboratory findings into effective stewardship programs.^13^

**Table 3.** Recommendations for enhancing AMR surveillance at the regional and national level

Recommendations for Enhancing AMR Surveillance at the Regional and National Level
1. Migrate the paper-based lab request form to an electronic version with mandatory fields to ensure capture of critical data, including unique patient and specimen identification. Ensure the form cannot be submitted unless these key fields are completed.
2. Establish a nationwide system for generating patient unique identifiers within the healthcare setting.
3. Standardize AMR data management applications nationwide and deploy ALIS at all locations that are not currently using it. This ensures uniform diagnostic workflows, enables complete, standardized data capture, and creates a streamlined, interoperable AMR data pipeline. Overall, this improves data quality, efficiency, and consistency nationwide.
4. Establish connectivity between ALIS and WHONET through BacLink and create an. ALIS export in WHONET to enable data transfer and analysis preparation.
5. Harmonize the AMR data collection using BacLink by establishing a national variable list and aligning BacLink configuration accordingly. This standardized BacLink configuration will capture all AMR data from the paper-based laboratory request form, AST results, and other relevant information nationwide.
6. Using the standardized national WHONET configuration, develop standard analytical reports for RRHs to support interventions. These customizable reports should include annual antibiograms, epidemiological trends, daily organism and resistance line lists by location with cluster alerts, and resistance profile analyses by organism, specimen source, location, cluster, and outbreak.
7. Develop macros for standardized reports to enhance analytical capacity, improve efficiency, and support long-term sustainability.
8. Integrate automation features in WHONET to streamline processes and improve access to analytical outputs.
9. Develop infrastructure within the healthcare system to facilitate the timely sharing of AMR products and alerts with stakeholders, utilizing tools such as network email or automated alert systems to support rapid feedback and intervention.
10. Develop standard operating procedures to guide implementation, support knowledge management, ensure training, and sustainability. Provide WHONET training, building on existing in-service programs to reinforce and refresh skills.
11. Establish a Centralized Data Integration and Sharing Information System that ensures AMR data is accessible at all levels and provides a comprehensive view of the country’s AMR status.
12. Establish processes and analytical capacity to detect clusters and outbreaks at the national level, and mechanisms for timely alerts and facility notifications.

Notes. ALIS, African Laboratory Information System; AMR, Antimicrobial Resistance; AST, Antimicrobial Susceptibility Testing; RRH, Regional Referral Hospital; WHONET, World Health Organization Network.

Findings from the simulation underscore the need to strengthen interoperability, digital infrastructure, and workforce capacity for effective AMR surveillance. Seamless integration between WHONET, ALIS, and ClinicMaster, coupled with automated data conversion and standardized operating procedures, can enhance analytical efficiency and sustainability. Standardized reports, such as antibiograms with customizable features, and structured use of WHONET macros further support consistency, knowledge transfer, and capacity building. Optimizing these digital and workforce systems is critical to translating AMR data into timely, actionable insights.

**Figure 3.**
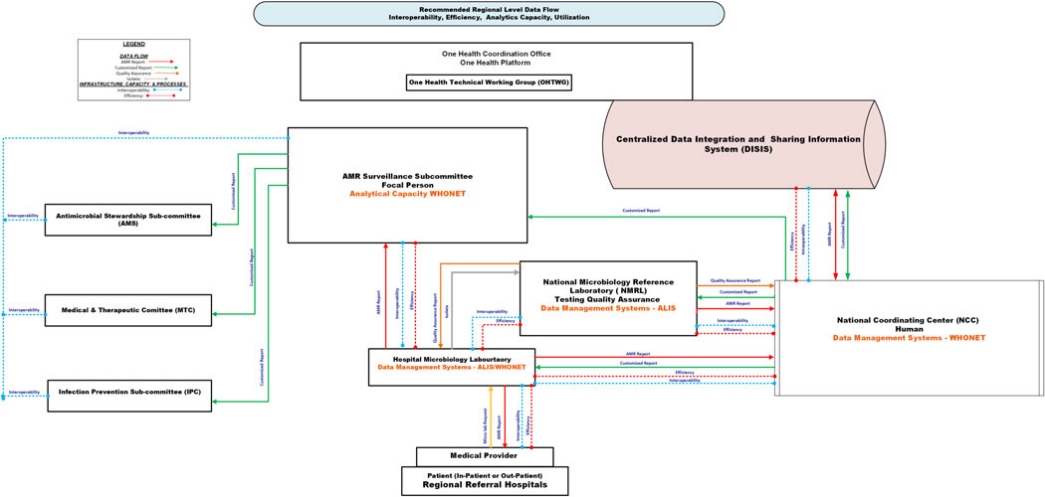
Schematic showing the recommended data flow at the regional level. This Figure depicts the recommended antimicrobial resistance (AMR) surveillance data flow from a regional level perspective. Solid arrows indicate key information flows: red = AMR surveillance reports from the World Health Organization Network (WHONET) or the African Laboratory Information System (ALIS); green = customized reports; orange = quality assurance feedback; grey = physical transfer of isolates. Dotted lines indicate infrastructure needs: blue = interoperability gaps; red = efficiency gaps. Data originates at the patient-provider interface and is captured at the hospital microbiology laboratory using WHONET or ALIS. Data are sent to the AMR focal person, who generates decision-support reports used by the AMR Surveillance Subcommittee, the Antimicrobial Stewardship Subcommittee (AMS), the Medicine and Therapeutic Committee (MTC), and the Infection Prevention and Control Subcommittee (IPC). AMR datasets and reports are transmitted to the National Microbiology Reference Laboratory (NMRL) for confirmatory testing and quality assurance using ALIS, and to the National Coordination Center (NCC) for national analysis and onward reporting. The NCC shares national AMR data with the Centralized Data Integration and Sharing Information System, which subsequently shares data with the One Health Technical Working Group (OHTWG). ALIS, African Laboratory Information System; WHONET, World Health Organization Network; NMRL, National Microbiology Reference Laboratory; NCC, National Coordination Center; OHTWG, One Health Technical Working Group; AMS, Antimicrobial Stewardship Subcommittee; MTC, Medicine and Therapeutic Committee; IPC, Infection Prevention and Control Subcommittee.

**Figure 4.**
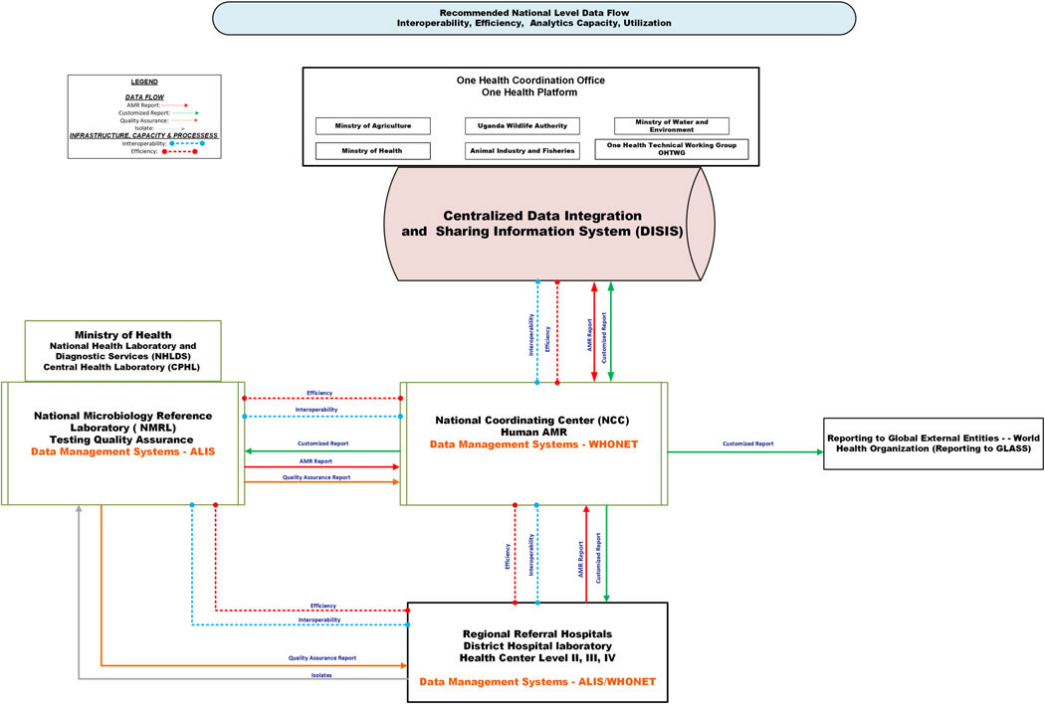
Schematic showing the recommended data flow at the national level. This Figure illustrates the recommended flow of antimicrobial resistance (AMR) data at the national level. Solid arrows represent key information flows: red = AMR surveillance reports from World Health Organization Network (WHONET) or African Laboratory Information System (ALIS); green = customized reports; orange = quality assurance feedback; grey = physical transfer of isolates. Dotted lines indicate infrastructure needs: blue = interoperability gaps; red = efficiency gaps. Data originates at the patient-provider interface and is captured at the hospital microbiology laboratory using WHONET or ALIS. AMR datasets and reports are transmitted to the National Microbiology Reference Laboratory (NMRL) for confirmatory testing and quality assurance, and to the National Coordination Center (NCC) for national analysis and reporting. The NCC shares AMR data with the Centralized Data Integration and Sharing Information System, which subsequently shares data with the One Health Technical Working Group (OHTWG). ALIS, African Laboratory Information System; WHONET, World Health Organization Network; NMRL, National Microbiology Reference Laboratory; NCC, National Coordination Center; OHTWG, One Health Technical Working Group.

Figures 3 and 4 depict the recommended regional and national AMR data flow, emphasizing the need for real-time access to decisional products for clinicians, infection control, and stewardship committees. Automated alert systems could strengthen feedback loops, enhancing timely responses to emerging resistance patterns.

Subsequent assessments should document implementation of these recommendations, scalability, and the impact of these enhancements on AMR surveillance and data utilization.

This study is limited by its reliance on interviews and process simulations, which may introduce bias or overlook specific barriers. The focus on a regional referral hospital limits generalizability to other settings. Nevertheless, triangulating perspectives from diverse stakeholders within the AMR system enhances confidence that the findings capture key system-level insights. The deliberate focus on human health, excluding the animal and environmental sectors, enables targeted recommendations to improve AMR interventions in this domain.
