# Enhancing antimicrobial resistance (AMR) surveillance data use in Uganda – ERRATUM

**DOI:** 10.1017/ash.2026.10372

**Published:** 2026-04-01

**Authors:** Uzo Chukwuma, Jonathan Mayito, Vivian Twemanye, Ritah Namusoosa, Dan Kakyakumaiso, Morgan Otita, Dickson Tabajjwa, Consolata Guma, David Brett-Major, Richard Walmema

The author’s name was incorrectly spelled in the article as originally published (David David Brett-Major). This has been corrected to David Brett-Major in the HTML and PDF versions of the article.

The publisher apologises for the error.
